# Association between probiotic therapy and the risk of hepatocellular carcinoma in patients with hepatitis B-related cirrhosis

**DOI:** 10.3389/fcimb.2022.1104399

**Published:** 2023-01-13

**Authors:** Ke Shi, Qun Zhang, Yi Zhang, Yufei Bi, Xuanwei Zeng, Xianbo Wang

**Affiliations:** Center of Integrative Medicine, Beijing Ditan Hospital, Capital Medical University, Beijing, China

**Keywords:** liver cancer, hepatitis B virus, probiotics, cirrhosis, propensity score matching, gut microbiota

## Abstract

**Objective:**

Probiotics may offer cancer-prevention benefits, based on experimental investigation results. This study aimed to determine the potential association between probiotics and hepatocellular carcinoma (HCC) in patients with hepatitis B-related cirrhosis (HBC) receiving antiviral therapy.

**Design:**

This retrospective study included 1267 patients with HBC treated with entecavir or tenofovir between January 2013 and December 2017. The risk of developing HCC was compared between two cohorts of 449 probiotic users (taking a cumulative defined daily doses [cDDD] of ≥ 28) and 818 non-probiotic users (< 28 cDDD). To eliminate the bias caused by confounding factors, propensity score matching (PSM) was used.

**Results:**

On multivariate regression analysis, probiotic consumption was an independent protective factor for HCC occurrence. After PSM, the incidence of HCC was significantly lower in the probiotic users than that in the nonusers (adjusted hazard ratio [aHR]: 0.70, 95% confidence interval: 0.59–0.83, *P* < 0.001). The aHRs for probiotics with 28–89, 90–180, and >180 cDDD were 0.58, 0.28, and 0.12, respectively, indicating a dose-response pattern. In 28–89, 90–180, and >180 cDDD, the 3-year cumulative incidence of HCC was 8.7%, 4.7%, and 3.0%, respectively. A multivariate stratified analysis confirmed that the administration of probiotics could help patients.

**Conclusion:**

Adjuvant probiotic therapy may reduce the risk of HCC in patients receiving antiviral medication for HBC. However, further clinical research is required to confirm these findings.

## Introduction

Hepatocellular carcinoma (HCC) is a severe health problem and has become the second leading cause of cancer mortality globally, accounting for 782,000 deaths annually (Bray et al., 2018). The hepatitis B virus (HBV) is the leading cause of cirrhosis and HCC worldwide (El-Serag, 2012). Cirrhosis affects approximately 80% of patients with a subsequent diagnosis of HCC. However, most patients are diagnosed at an advanced stage, with limited treatment options and a low survival rate (Balogh et al., 2016). Current antiviral therapies, such as nucleos(t)ide analogs [NA(s)], are efficacious in inhibiting viral replication, improving liver inflammation, and reducing HCC incidence (Chang et al., 2010; Marcellin et al., 2013). Several studies have reported that antiviral therapy can attenuate, but not completely eliminate the risk of HCC in patients with hepatitis B-related cirrhosis (HBC) (Lee and Ahn, 2016). Despite HBV replication decrease, antiviral therapy alone may be insufficient to prevent HCC. Because of the growing incidence and high mortality rate of HCC, new treatments or prophylactic strategies are urgently needed to lower the risk of HCC.

Probiotics, as live microorganisms, provide health benefits to the host when administered in adequate amounts (Wan and El-Nezami, 2018). Recent studies have reported that probiotics could be used as an alternative for cancer prevention and treatment (Yu and Li, 2016; Singh et al., 2021). Probiotics can regulate the intestinal microflora and maintain the balance in the microbiota ecosystem (Mendoza, 2019). Probiotics influence various biological processes associated with cancer, such as inflammation, oxidative stress, apoptosis, proliferation, and metastasis (Thilakarathna et al., 2021). In addition, probiotics are reported to play an important role in cancer prevention by regulating the microbiome and immune response (Feng et al., 2018). Therefore, clinical trials on the modulation of microbiota dysbiosis by probiotics will contribute to the clinical application of probiotics in the future management of several cancer types.

Multiple researchers have reported that patients with HBC have an altered gut microbiota and significantly greater plasma endotoxin levels than healthy individuals (Qin et al., 2014; Chen et al., 2021). Microbial dysbiosis and endotoxemia are important contributing factors to cirrhosis in HCC (Wan and El-Nezami, 2018). Probiotics have an antiviral activity that slows HCC progression by preventing chronic HBV infection (Lee et al., 2013; Thilakarathna et al., 2021). In an animal study, VSL#3 treatment reduced liver inflammation and restored intestinal homeostasis, preventing cirrhosis progression to HCC (Zhang et al., 2012; Li et al., 2016). Considering the increasing use of probiotics and incidence of HCC, the potential link between probiotics and HCC is an important issue to investigate. However, few studies have reported the effects of adjuvant probiotic therapy on the risk of HCC in patients with HBC in clinical practice. Propensity score matching (PSM) has been used to reduce bias caused by potential confounding factors between groups (Rusthoven et al., 2016; Tian et al., 2022). PSM is an effective statistical method to explore the efficacy of probiotics and HCC occurrence in patients with HBC.

Therefore, we used PSM to investigate the association between probiotic therapy and the risk of HCC among patients with HBC and to provide clinical evidence for probiotics as adjuvant therapy in these populations.

## Materials and methods

### Participants

We examined 1932 patients treated for HBC at Beijing Ditan Hospital, Capital Medical University, between January 2013 and December 2017. Patients with HBC aged 20–75 years were enrolled in this study. Exclusion criteria were being infected with human immunodeficiency virus; having other hepatitis infections, malignancies, or liver failure; having undergone liver transplantation; having alcoholic liver and severe fatty liver; having obtained a probiotic prescription before the index date; and having died or had less than 3 years of follow-up. After applying the exclusion criteria, 1267 patients were enrolled in the study.

**Figure 1:**
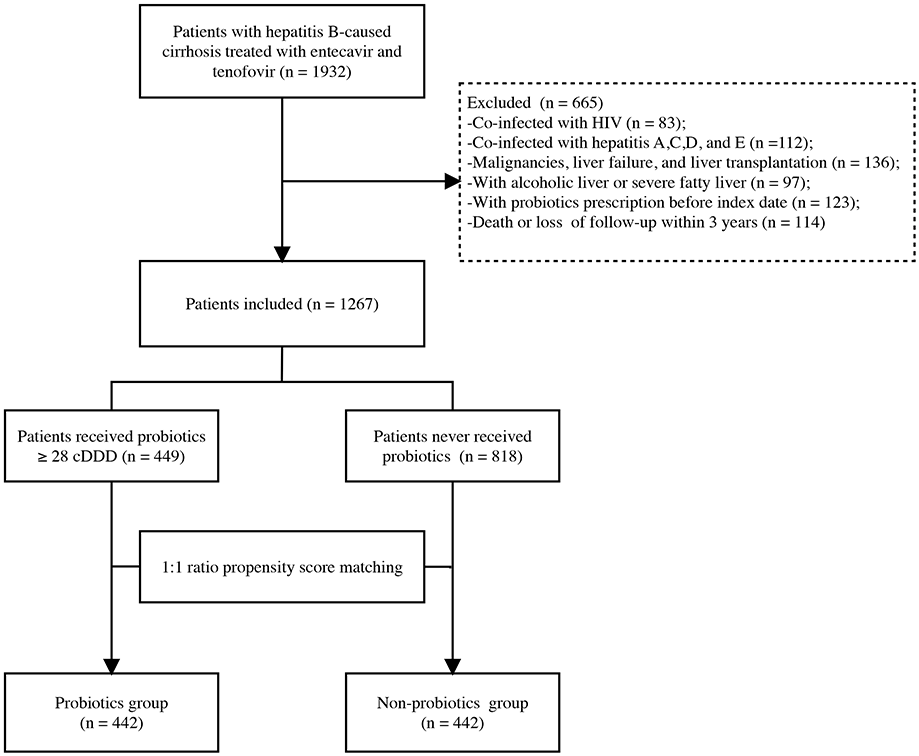
Flowchart of the enrollment of patients in the study. HCC, hepatocellular carcinoma; HIV, human immunodeficiency virus; cDDD, cumulative defined daily doses.

Patients were divided into two cohorts based on the clinically effective proportion of probiotic therapy they received: probiotics ≥ 28 cumulative defined daily doses (cDDD) based on antiviral therapy or no probiotic at all (Marlicz et al., 2016). Ultimately, 449 patients received probiotics ≥ 28 cDDD, and 818 patients did not receive probiotics. Thus, 442 probiotic users and 442 non-users were randomly paired after a 1:1 PSM (**Figure 1**). The index date was the date of the diagnosis of cirrhosis at our hospital. The outcome of this study was the occurrence of HCC or the end of the 3-year follow-up period. The study protocol was performed in accordance with the principles of the Declaration of Helsinki and was approved by the ethics committee of Beijing Ditan Hospital.

### Clinical definitions and data collection

Chronic hepatitis B is defined as the persistent presence of the hepatitis B surface antigen for > 6 months (Sarin et al., 2016). Cirrhosis was diagnosed based on the following criteria: liver biopsy; endoscopic or ultrasound abnormal findings; cirrhotic elastography; and complications related to portal hypertension, such as ascites, hepatic encephalopathy (HE), and esophagogastric variceal bleeding (Shiha et al., 2009). Histological or radiological evidence (computed tomography or magnetic resonance imaging) was used to diagnose HCC (Bruix and Sherman, 2011). DDD was used to measure the prescribed dose of probiotics as recommended by the World Health Organization (WHO) (WHO Collaborating Center for Drugs Statistics Methodology, 2003). For example, the average daily maintenance dose of probiotics for an adult is 1 DDD. The cDDD, which indicates the duration of administration of probiotics, is the sum of all cDDDs administered during the 3-year follow-up. The probiotic group received at least 28 cDDD of probiotics after diagnosis of HBC (based on data from the medical records of probiotics used at our hospital). Virological response (VR) was defined as an undetectable HBV DNA load at the end of the study.

Baseline demographic variables and laboratory measurements, including hemogram; liver, renal, and coagulation tests; HBV DNA; and alpha-fetoprotein (AFP), were recorded from a computerized database during the first week of enrollment. The model for end-stage liver disease (MELD) and Child-Turcotte-Pugh (CTP) scores were used to estimate the severity of the liver disease (Wiesner et al., 2003; Botero and Lucey, 2003). Every 3–6 months, routine laboratory tests and radiological examinations were performed. The probiotic dosage, frequency, total number of prescriptions, and duration were recorded during the study period.

### Treatments

Patients with HBC received first-line antiviral therapy, such as entecavir (ETV) or tenofovir (TDF). Moreover, according to the doctor’s diagnosis, some patients received probiotics as an additional medication to balance the gut flora. Prescription drugs are included in our database by name, dose, and course of treatment. *Bacillus licheniformis* capsule; live combined *Bacillus subtilis* and *Enterococcus faecium* enteric-coated capsules; live combined *Bifidobacterium*, *Lactobacillus*, and *Enterococcus* capsules; and live combined *Bifidobacterium*, *Lactobacillus*, *Enterococcus*, and *Bacillus cereus* tablets were included in this study. **Table S1** shows more information on the probiotics used in this study.

### Statistical analysis

The t-test was used to compare normally distributed continuous variables, whereas the Mann–Whitney U test was used for non-normally distributed variables. The chi-square or Fisher’s exact test was used to evaluate categorical variables. To identify independent risk factors influencing 3-year HCC occurrence, univariate and multivariate Cox proportional hazard regression models were used. The propensity score is the probability of treatment assignment conditional on baseline characteristics (Austin, 2011). Using the propensity score, the distribution of baseline characteristics between exposed and unexposed patients is independent of the therapy received, which allows for the estimation of unbiased treatment effects (Austin, 2011). A logistic regression model was used to calculate PSM based on variables related to outcome to balance the potential bias and minimize confounding variables. The probiotics users and nonusers were matched randomly by age, sex, ascites, HE, alanine aminotransferase (ALT), platelet count, and NA(s) treatment before ETV/TDF. The nearest neighbor matching algorithm with a caliper width of 0.05 was used to undertake one-to-one matching without replacement. The caliper width was 0.05 times the standard deviation of the logit of the propensity score (Austin, 2011), which eliminate at least 99% of the bias due to confounding factors (Rusthoven et al., 2016). The performance of the propensity score model was assessed by evaluating whether any important relationships between the exposure groups and the covariates remained after adjustment. SPSS 25.0 (IBM Corp., Armonk, New York, USA) was used for the analyses.

To examine the dose-response relationship, we divided patients into four groups according to probiotics use: 28–89, 90–180, and >180 cDDD. The Kaplan-Meier method was employed to estimate the cumulative incidence of HCC, and the log-rank test was utilized to assess the differences between the curves. The process was generated using the packages of survival *via* R software (version 4.1.3; The R Foundation, Vienna, Austria). The Cox proportional hazards regression model was used to calculate the multivariate-adjusted hazard ratios (aHRs) and 95% confidence intervals (CIs) of HCC associated with probiotic administration. Additionally, as a sensitivity analysis, a multivariate stratified analysis was performed to investigate the difference and consistency between probiotic therapy and the risk of HCC in the patient subgroups. A two-sided *P* < 0.05 was considered statistically significant. The supporting data and related scripts are available on GitHub (https://github.com/Shike130909/Probiotic-therapy-and-the-risk-of-HCC).

## Results

### Baseline characteristics

Of the 1267 patients with HBC, 134 (10.6%) developed HCC during the 3-year follow-up. **Table 1** shows the baseline characteristics and laboratory variables for both the unmatched and matched cohorts. Before PSM, patients in the probiotics group had higher total bilirubin, platelet levels, and longer prothrombin time than those in the non-probiotics group (all *P* < 0.05). In the probiotic group, patients had a significant percentage of ascites and HE. Moreover, probiotic users had higher MELD and CTP scores than nonusers (*P* < 0.001). The VR was achieved in 97% and 98.7% of the probiotic and non-probiotics groups at the end of 1 year after antiviral therapy, respectively. After PSM, the two groups’ demographic and clinical baseline characteristics were consistent, and a matched cohort of 884 patients was included in the analysis.

**Table 1:** Clinical characteristics of patients with hepatitis B-related cirrhosis in unmatched and matched cohorts.

Variables	Unmatched cohort			Matched cohort		
	Probiotics user (n = 449)	Non-probiotics users (n = 818)	*P* value	Probiotics user (n = 442)	Non-probiotics users (n = 442)	*P* value
Age, years	51.1 ± 10.2	49.7 ± 10.8	**0.020**	51.1 ± 10.7	50.3 ± 10.9	0.135
Male sex, n (%)	305 (67.9%)	588 (71.9%)	0.140	301 (68.1%)	312 (70.6%)	0.422
Family history of HCC, n (%)	37 (8.2%)	66 (8.1%)	0.915	38 (8.7%)	25 (5.7%)	0.089
Alcohol use, n (%)	104 (23.2%)	187 (22.9%)	0.903	103 (23.3%)	73 (16.5%)	0.012
Smoking, n (%)	107 (23.8%)	205 (25.1%)	**0.627**	106 (24.0%)	96 (21.7%)	0.423
Variceal bleeding, n (%)	86 (19.2%)	150 (18.3%)	0.721	85 (19.2%)	90 (20.4%)	0.673
Ascites, n (%)	189 (42.1%)	273 (33.4%)	**0.001**	185 (41.9%)	186 (42.1%)	0.946
Hepatic encephalopathy, n (%)	30 (6.7%)	32 (3.9%)	**0.029**	26 (5.9%)	21 (4.8%)	0.454
Alanine aminotransferase, U/L	35.6 (24.2, 81.7)	37.3 (24.3, 64.5)	0.442	36.2 (24.6, 81.2)	37.3 (24.3, 84.2)	0.627
Aspartate aminotransferase, U/L	47.0 (32.1, 84.1)	42.4 (28.9, 67.1)	0.784	47.0 (32.2, 84.5)	44.7 (30.0, 87.0)	0.926
Total bilirubin, μmol/L	28.5 (19.3, 46.7)	22.4 (14.5, 36.8)	**0.016**	28.4 (19.2, 46.1)	24.5 (15.5, 41.5)	0.400
Albumin, g/L	31.2 ± 6.0	33.6 ± 6.7	**< 0.001**	31.3 ± 6.0	32.5 ± 6.2	0.078
White blood cell count, ×10^9^/L	3.6 (2.7, 4.9)	4.0 (2.8, 5.6)	**0.001**	3.6 (2.6, 4.9)	3.7 (2.6, 5.3)	0.134
Platelet count, ×10^9^/L	71.6 (48.0, 87.2)	69.5 (48.0,102.5)	**0.006**	64.6 (46.0, 87.0)	64.6 (45.3, 94.5)	0.573
Creatinine, μmol/L	64.0 (55.0, 74.0)	66.0 (58.0, 75.9)	0.441	64.0 (55.0, 74.0)	66.0 (56.1, 75.0)	0.615
Blood urea nitrogen, mmol/L	5.1 (3.9, 6.7)	5.3 (4.4, 6.8)	0.385	5.1 (3.9, 6.8)	5.3 (4.1, 6.8)	0.892
Prothrombin time (s)	15.2 (13.8, 17.1)	14.5 (13.0, 16.3)	**0.001**	15.2 (13.8, 17.0)	15.0 (13.3, 16.8)	0.159
International normalized ratio	1.3 (1.1, 1.4)	1.2(1.1, 1.4)	0.865	1.2 (1.1, 1.4)	1.2(1.1, 1.4)	0.494
Alpha-fetoprotein, ng/ml	6.5 (3.2, 25.4)	7.4 (3.3, 34.9)	0.166	6.5 (3.2, 25.4)	8.0 (3.9, 35.4)	0.082
HBV DNA, log_10_IU/ml	3.5 (2.7, 5.6)	4.4 (2.7, 5.8)	0.737	4.4 (2.7, 5.6)	4.6 (2.7, 5.6)	0.180
NA(s) treatment before ETV/TDF, n (%)	123 (27.4)	238 (29.1)	0.521	121 (27.4)	104 (23.5)	0.189
Child-Pugh score	9.0 (7.0, 10.0)	8.0 (6.0, 9.0)	**< 0.001**	9.0 (7.0, 10.0)	8.0 (6.0, 10.0)	0.057
MELD score	8.6 (7.3, 11.5)	7.9 (6.4, 10.2)	**< 0.001**	8.6 (7.3, 11.3)	8.6 (6.6, 11.1)	0.491

MELD, Model for End-Stage Liver Disease. Bold values: P < 0.05.

### Analysis of risk factors

As presented in **Table 2**, in the multivariate Cox regression analysis that was adjusted for age, sex, family history of HCC, ascites, HE, ALT, albumin, platelets, blood urea nitrogen, HBV DNA, and NA(s) treatment before ETV/TDF, probiotics use was still an independent factor against HCC occurrence (aHR = 0.43, 95% CI 0.28–0.65; *P* < 0.001). Older age (aHR = 1.04, 95% CI 1.02–1.06; *P* < 0.001), family history of HCC (aHR = 1.83, 95% CI 1.13–2.96; *P* = 0.014), ascites (aHR = 1.76, 95% CI 1.06–2.92; *P* = 0.028), HE (aHR = 2.62, 95% CI 1.45–4.71; *P* = 0.001), and higher blood urea nitrogen (aHR = 1.05, 95% CI 1.00–1.10; *P* = 0.048) also significantly increased HCC risk. However, using NA(s) treatment before ETV/TDF was related to a lower HCC risk (aHR = 0.50, 95%CI 0.32–0.78; *P* = 0.002).

**Table 2:** Cox proportional hazards regression model analysis for risk of HCC in patients with hepatitis B-related cirrhosis.

Variables	Univariate analysis		Multivariate analysis	
	HR (95% CI) *P* value		HR (95% CI) *P* value	
Probiotics use	0.51 (0.34–0.76)	**< 0.001**	0.46 (0.31–0.70)	**< 0.001**
Age (years)	1.04 (1.03–1.06)	**< 0.001**	1.04 (1.02–1.05)	**< 0.001**
Sex (male vs. female)	0.91 (0.62–1.33)	0.637		
Family history of HCC	2.09 (1.30–3.36)	**0.002**	1.83 (1.13–2.96)	**0.014**
Alcohol consumption	1.25 (0.85–1.83)	0.251		
Diabetes	1.35 (0.72–1.77)	0.583		
Variceal bleeding	1.23 (0.82–1.86)	0.320		
Ascites	1.85 (1.32–2.60)	**0.001**	1.76 (1.06–2.92)	**0.028**
Hepatic encephalopathy	2.52 (1.45–4.38)	**0.001**	2.62 (1.45–4.71)	**0.001**
Alanine aminotransferase (U/L)	0.99 (0.99–0.99)	**< 0.001**	0.99 (0.99–0.99)	**0.004**
Aspartate aminotransferase (U/L)	0.99 (0.99–1.00)	0.055		
Total bilirubin (μmol/L)	0.99 (0.99–1.01)	0.525		
Albumin (g/L)	0.97 (0.95–0.99)	**0.042**		
White blood cell count (×10^9^/L)	1.05 (0.98–1.13)	0.149		
Platelet count (×10^9^/L)	0.99 (0.98–0.99)	**< 0.001**	0.99 (0.98–0.99)	**0.004**
Creatinine (mg/dl)	1.00 (0.99–1.01)	0.480		
Blood urea nitrogen (mg/dl)	1.06 (1.02–1.12)	**0.010**	1.05 (1.00–1.10)	**0.048**
Prothrombin time (s)	1.02 (0.97–1.07)	0.454		
Alpha-fetoprotein (ng/ml)	1.00 (0.99–1.01)	0.820		
HBV DNA (log_10_IU/ml)	1.13 (1.03–1.24)	**0.010**		
NA(s) treatment before ETV/TDF	0.54 (0.35–0.84)	**0.006**	0.50 (0.32–0.78)	**0.002**
Child-Pugh score	1.07 (0.99–1.14)	0.060		
MELD score	1.01 (0.96–1.06)	0.656		

NA(s), nucleos(t)ide analogue(s); ETV, entecavir; TDF, tenofovir; MELD, Model for End-Stage Liver Disease. Bold values: P < 0.05.

### Effects of probiotics on HCC occurrence

**Figure 2:**
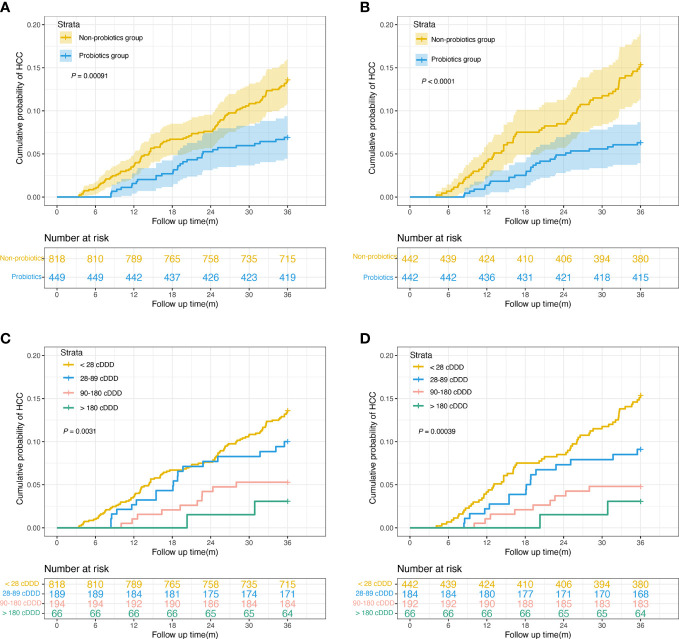
Cumulative incidence of HCC in patients with HBC. **(A)** Cumulative incidence of HCC among probiotics (n = 449) and nonusers (n = 818) in the unmatched cohort (6.7% vs. 12.7%, log-rank test *P* = 0.00091). **(B)** Cumulative incidence of HCC among probiotics (n = 442) and nonusers (n = 442) in the matched cohort (6.1% vs. 14.0%, log-rank test *P* < 0.0001). **(C)** Cumulative incidence of HCC in patients with < 28 (n = 818), 28–89 (n = 189), 90–180 (n = 194), and > 180 cDDD (n = 66) of probiotics use before matching (12.7% vs. 9.5% vs. 5.2% vs. 3.0%, log-rank test *P* = 0.0031). **(D)** Cumulative incidence of HCC in patients with with < 28 (n = 442), 28–89 (n = 184), 90–180 (n = 192), and > 180 cDDD (n = 66) of probiotics use after matching (14.0% vs. 8.7% vs. 4.7% vs. 3.0%, log-rank test *P* = 0.00039). HCC, hepatocellular carcinoma; HBC, hepatitis B-related cirrhosis; cDDD, cumulative defined daily doses.

In the unmatched cohort, the probiotics group had a significantly lower incidence of HCC than the non-probiotics group (6.7% vs. 12.7%, *P* < 0.001; **Figure 2A**). After PSM, the 3-year HCC occurrence rates in probiotic users and nonusers were 6.1% and 14.0%, respectively (*P* < 0.001; **Figure 2B**). The probiotics group was categorized into three subgroups based on the probiotics dose used: 28–89 cDDD, 90–180 cDDD, and > 180 cDDD. **Figures 2C, D** show the Kaplan–Meier analysis results for the unmatched cohort and matched cohort. The probiotics group had a significantly reduced incidence of HCC in a dose-response manner than the non-probiotics group (both *P* < 0.005).

Additionally, **Table 3** shows the dose-response relationship between the administration of probiotics and the risk of HCC development. In the unmatched cohort, the aHRs were 0.67 (95% CI, 0.41–1.11, *P* = 0.120), 0.31 (95% CI, 0.16–0.58, *P* = 0.001), and 0.15 (95% CI, 0.04–0.61, *P* = 0.008) for patients that received 28–89, 90–180, and > 180 cDDD, probiotics respectively. In the matched cohort, the overall aHR for HCC development was 0.59 (95% CI, 0.34–0.81, *P* < 0.001) for probiotic users versus non-probiotic users. The aHRs for probiotic usage of 28–89 cDDD, 90–180 cDDD, and > 180 cDDD, were 0.58 (95% CI, 0.33–1.01, *P* = 0.053), 0.28 (95% CI, 0.14–0.56, *P* = 0.001), and 0.12 (95% CI, 0.03–0.52, *P* = 0.004), respectively.

**Table 3:** Risk of of hepatocellular carcinoma according to probiotics use in unmatched and matched cohorts.

Strata	Total	HCC	Crude HR (95%CI)	Adjusted HR (95%CI)
Unmatched cohort				
Non–probiotics user (< 28cDDD)	818	104	1 (Reference)	1 (Reference)
Probiotics user (≥ 28cDDD)	449	30	0.45 (0. 29–0.72) *P <*0.001	0.69 (0.60–0.81) *P* < 0.001
28–90 cDDD	189	18	0.74 (0.45–1.21) *P* = 0.235	0.67 (0.41–1.11) *P* = 0.120
90–180 cDDD	194	10	0.39 (0.20–0.74) *P* = 0.004	0.31 (0.16–0.58) *P* = 0.001
>180 cDDD	66	2	0.22 (0.05–0.91) *P* = 0.037	0.15 (0.04–0.61) *P* = 0.008
Matched cohort				
Non–probiotics user (< 28cDDD)	442	63	1 (Reference)	1 (Reference)
Probiotics user (≥ 28cDDD)	442	27	0.70 (0.59–0.83) *P* < 0.001	0.59 (0.34–0.81) *P* < 0.001
28–90 cDDD	184	16	0.59 (0.34–1.03) *P* = 0.064	0.58 (0.33–1.01) *P* = 0.053
90–180 cDDD	192	9	0.31 (0.15–0.63) *P* = 0.001	0.28 (0.14–0.56) *P* = 0.001
>180 cDDD	66	2	0.20 (0.05–0.82) *P* = 0.025	0.12 (0.03–0.52) *P* = 0.004

Adjusted HR represents multivariate-adjusted hazard ratio: age, sex, Family history of HCC, ascites, hepatic encephalopathy, alanine aminotransferase, platelet count, NA(s) treatment before ETV/TDF. HR, hazard ratio; cDDD, cumulative defined daily doses; CI, confidence interval; HCC, hepatocellular carcinoma; NA(s), nucleos(t)ide analogue(s); ETV, entecavir; TDF, tenofovir.

### HCC risk analyses using MELD score and CTP class

**Figure 3:**
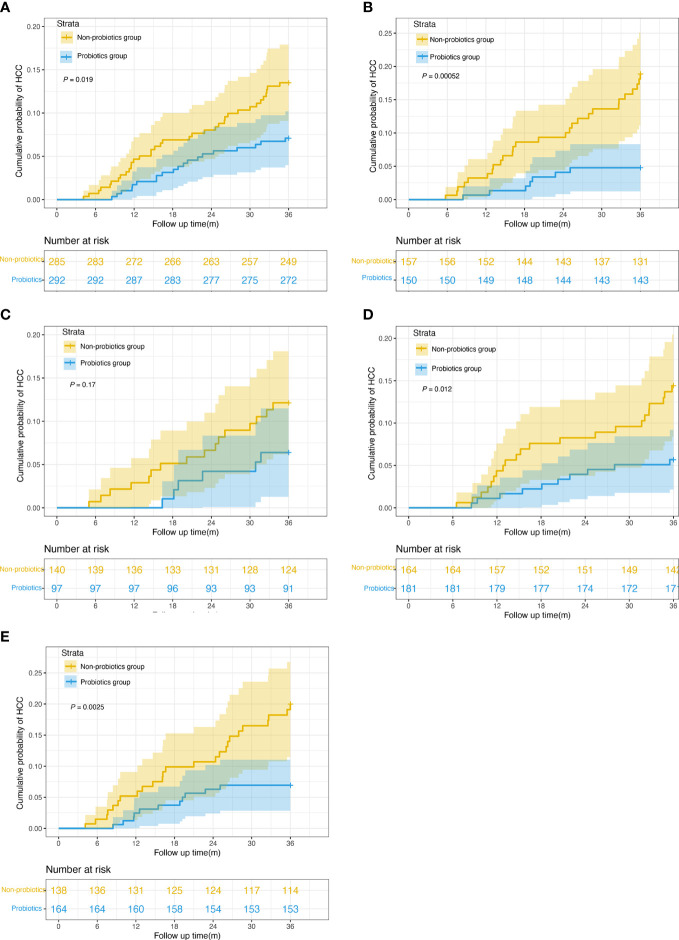
Kaplan-Meier survival curves of risk stratification according to MELD score and CTP classification. **(A)** Cumulative incidence of HCC in probiotics users and nonusers with MELD scores < 10 (n = 577, 6.8% vs. 12.6%, log-rank test *P* = 0.019). **(B)** Cumulative incidence of HCC in probiotics users and nonusers with MELD scores ≥ 10 (n = 307, 4.7% vs. 16.5%, log-rank test *P* = 0.00052). **(C)** Cumulative incidence of HCC in probiotics users and nonusers in CTP class A (n = 237, 6.2% vs. 11.4%, log-rank test *P* = 0.17). **(D)** Cumulative incidence of HCC in probiotics users and nonusers in CTP class A (n = 345, 5.5% vs. 13.4%, log-rank test *P* = 0.012). **(E)** Cumulative incidence of HCC in probiotics users and nonusers in CTP class A (n = 302, 6.7% vs. 17.4%, log-rank test *P* = 0.0025). MELD, Model for End-Stage Liver Disease. CTP,Child-Turcotte-Pugh.

After PSM, 577 patients had MELD scores <10, and 307 patients had MELD scores ≥10. When the MELD was <10, the 3-year HCC incidences were 12.6% and 6.8% in the non-probiotic and probiotics groups, respectively (*P* = 0.019, **Figure 3A**). When the MELD was ≥10, the 3-year HCC incidences were 16.5% and 4.7% in the non-probiotics and probiotics groups, respectively (*P* < 0.001, **Figure 3B**). Additionally, 237 patients belonged to CTP class A; 345, to CTP class B; and 302, to CTP class C. The 3-year HCC incidences were 11.4% and 6.2% in CTP class A (*P* = 0.17, **Figure 3C**), 13.4% and 5.5% in CTP class B (*P* = 0.012, **Figure 3D**), and 17.4% and 6.7% in CTP class C (*P* = 0.0025, **Figure 3E**) in the non-probiotics and probiotics groups, respectively.

### Multivariate stratified analysis for probiotic therapy

The multivariate stratified analysis was used to investigate the association between the administration of probiotics and the risk of HCC in patient subgroups (**Table 4**). We examined the outcome in groups based on age, sex, ascites, HE, ALT level, platelet counts, and NA(s) treatment before ETV/TDF. Among patients aged 50 years or younger (HR: 0.31, 95% CI 0.11–0.83), older than 50 years (HR: 0.42, 95% CI 0.25–0.69); males (HR: 0.40, 95% CI 0.23–0.66); and those with (HR: 0.36, 95% CI 0.19–0.67) or without ascites (HR: 0.48, 95% CI 0.24–0.93), without HE (HR: 0.43, 95% CI 0.27–0.68), with ALT levels < 40 U/L (HR: 0.49, 95% CI 0.28–0.85) or ≥ 40 U/L (HR: 0.29, 95% CI 0.13–0.65), with platelet counts < 80 ×10^9^/L (HR: 0.39, 95% CI 0.22–0.67), and without NA(s) treatment before ETV/TDF (HR: 0.38, 95% CI 0.23–0.62), the positive effects of administration of probiotics continued. Although the HR was < 1.0 for most subgroups, statistical significance was not reached in several subgroups, including females (HR: 0.48, 95% CI 0.19–1.21), those with HE (HR: 0.24,95% CI 0.05–1.16) and platelet counts ≥ 80 ×10^9^/L (HR: 0.46,95% CI 0.25–1.00), and those with NA(s) treatment before ETV/TDF (HR: 0.85, 95% CI 0.24–2.94).

**Table 4:** Multivariable stratified analyses of the association between probiotics use and HCC occurrence.

Category	Probiotic users	Non-probiotic users	HR (95% CI)	*P* value
	HCC/Total (n, %)	HCC/Total (n, %)		
Age (years)				
< 50	5/180 (2.8%)	18/205 (8.8%)	0.31 (0.11–0.83)	**0.020**
≥ 50	22/262 (8.4%)	45/237 (18.9%)	0.42 (0.25–0.69)	**< 0.001**
Sex				
Male	20/301 (6.6%)	50/312 (16.0%)	0.40 (0.23–0.66)	**< 0.001**
Female	7/141 (4.9%)	13/130 (10.0%)	0.48 (0.19–1.21)	0.122
Ascites				
Yes	14/185 (7.6%)	37/186 (19.8%)	0.36 (0.19–0.67)	**< 0.001**
No	13/257 (5.0%)	26/256 (10.1%)	0.48 (0.24–0.93)	**0.032**
HE				
Yes	2/26 (7.7%)	6/21 (28.5%)	0.24 (0.05–1.16)	0.075
No	25/416 (6.0%)	57/421 (13.5%)	0.43 (0.27–0.68)	**< 0.001**
ALT (U/L)				
< 40	19/245 (7.7%)	36/237 (15.2%)	0.49 (0.28–0.85)	**0.012**
≥ 40	8/197 (4.1%)	27/205 (13.2%)	0.29 (0.13–0.65)	**0.003**
Platelet count (×10^9^/L)				
< 80	18/299 (6.0%)	43/289 (14.9%)	0.39 (0.22–0.67)	**0.001**
≥ 80	9/143 (6.3%)	20/153 (13.1%)	0.46 (0.25–1.00)	0.052
NA(s) treatment before ETV/TDF				
Yes	5/121 (4.1%)	5/104 (4.8%)	0.85 (0.24–2.94)	0.361
No	22/321 (6.8%)	58/338 (17.1%)	0.38 (0.23–0.62)	**< 0.001**

HCC, hepatocellular carcinoma; HE, hepatic encephalopathy; ALT, alanine aminotransferase.NA(s), nucleos(t)ide analogue(s); ETV, entecavir; TDF, tenofovir. Bold values: P < 0.05.

## Discussion

HCC is commonly associated with cirrhosis, and early detection is an excellent way to enhance patients’ longevity. The gut microbiota is an essential intestinal ecosystem; its dysbiosis can disrupt intestinal homeostasis and lead to harmful bacterial overgrowth, causing liver fibrosis, cirrhosis, and HCC. Growing evidence suggests that microbial imbalance causes cancer *via* various pathways involving inflammation and immunological dysregulation (Plottel and Blaser, 2011; Zhang et al., 2020a). Probiotics are new adjuvant therapies to prevent and reduce the risk of HCC (Wan and El-Nezami, 2018). Endotoxemia and the number of bacterial species can be reduced by administering *Bifidobacteria* and *Lactobacilli* (Tandon et al., 2009; Rincón et al., 2014). In this study, we observed a significant inverse association between probiotics use and the risk of HCC in patients with HBC on antiviral therapy. Furthermore, the administration of probiotics reduced the incidence of HCC in a dose-dependent manner.

Several studies have found a close association between gut microbiota alterations and HBC and HCC. Studies have shown that probiotics reduce the length of hospital stay, improve portal hypertension and associated complications, and lower inflammatory factors while causing no adverse events (Dhiman et al., 2014; Rincón et al., 2014). Herein, probiotics were found to be an independent protective factor against HCC in patients with HBC. Probiotic use was associated with a significantly lower risk of HCC, regardless of whether PSM was performed (both *P* < 0.001). Additionally, the risk reduction of HCC showed a dose-response relationship in the unmatched and matched cohorts. The uncorrected HR for overall probiotic prescription after PSM was 0.70 (95% CI: 0.59–0.83), while the aHR was 0.59 (95% CI: 0.34–0.81). With non-probiotics users as controls, the aHRs were 0.58 (95% CI: 0.33–1.01), 0.28 (95% CI: 0.14–0.56), and 0.12 (95% CI: 0.03–0.52) for 28–89, 90–180, and >180 cDDD, respectively, In a recent study, the high-dose probiotic VSL#3 reduced the risk of lesions in mice as compared to low-dose probiotics or no therapy (Li et al., 2016), indicating that HCC risk decreased as the cumulative dose of probiotics increased. Therefore, probiotic therapy may improve prognosis by reducing HCC morbidity and mortality.

The association between microbiota and the host plays a crucial role in the pathogenesis of HCC. Altering the composition of the intestinal flora can limit bacterial translocation, promote gut microflora balance, and prevent HCC progression (Zhang et al., 2012b). Sandler et al. reported that liver disease severity is related to bacterial translocation and overgrowth (Sandler et al., 2011). In the present study, probiotic users benefited significantly, especially patients with CTP B and C classes. A similar effect of probiotics use was demonstrated in our previous study in patients with cirrhosis (Zhang et al., 2020b). The MELD score is a parameter of a liver function used to assess the prognosis of patients with cirrhosis (Bernardi et al., 2011). Probiotics users had a lower HCC risk than non-probiotics users, regardless of MELD score < 10 or ≥ 10. However, the statistical difference between the two groups was more pronounced when the MELD score was ≥ 10. Preveden et al. previously showed that changes in the gut microflora are affected by liver disease stage (Preveden et al., 2017). Patients with advanced MELD and CTP scores show greater perturbations in microbial toxins, lipopolysaccharide secretion, and proinflammatory cytokine levels (Rayes et al., 2002; Rayes et al., 2005). Therefore, these patients were positively correlated with the benefit.

Probiotic therapy is a novel regimen for chronic liver diseases complicated by gut dysbiosis. Hepatocarcinogenesis is aided by gut microbial dysbiosis. Accumulating evidence has suggested that the gut–liver axis plays a key role in the progression of cirrhosis and HCC (Wiest et al., 2017; Ohtani and Kawada, 2019). Progress has been made in understanding the relationship between microbiota dysbiosis and carcinogenesis. Several experimental studies have reported that probiotics positively impact hosts (Patel and DuPont, 2015; Elzouki, 2016). The mechanism by which probiotics increase HCC risk may be associated with the following: (1) Gut microbiota modulation. The production of short-chain fatty acids (SCFAs) such as butyrate may reverse intestinal dysbiosis and reduce the risk of HCC (Li et al., 2016). Some studies reported that probiotics prevent carcinogenesis, by producing SCFAs, controlling gut microbiota composition, and improving gut barrier integrity (LeBlanc et al., 2017). (2) Improvement of gut barrier integrity. Gut bacterial dysbiosis damages the intestinal barrier, promotes bacterial translocation, and, subsequently, induces inflammatory responses (Zhang et al., 2012; Bajaj et al., 2018). Probiotics can enhance the intestinal barrier and decrease inflammation, thereby inhibiting HCC progression (Zhang et al., 2012). (3) Immune regulation and anti-inflammatory role. Gut microbial dysbiosis triggers a pro-inflammatory immune response and cancer progression (Singh et al., 2021). A study has reported that probiotics promote the growth of beneficial microbes, producing anti-inflammatory metabolites with antitumor activity (Li et al., 2016). (4) Antimicrobial effect. Bacterial translocation leads to endotoxemia, which results in portal hypertension and liver cell damage, thus contributing to HCC occurrence (Tao et al., 2015). Probiotics can release antimicrobial molecules, reduce bacterial translocation and overgrowth, and improve endotoxemia (Seki and Schnabl, 2012; Petschow et al., 2013). Thus, these possible mechanisms explain how probiotics reduce the risk of HCC in patients with HBC.

To the best of our knowledge, this is the first observational study to investigate the benefits of probiotics in reducing HCC risk in a population-based cohort of patients with HBC on antiviral therapy. However, this study had several limitations. First, this was an observational retrospective study; thus, potential confounding risk factors may have existed. Despite careful PSM, we cannot exclude the possibility of residual confounding resulting from unmeasured variables, such as diet, coffee, duration of antiviral therapy, and course of cirrhosis. Further prospective randomized controlled trials are required to determine the impact of these risk factors on developing HCC. Second, owing to the single-center study design, the number of patients was limited. Large-sample, multicenter studies need to be conducted in the future. Third, due to the cross-use of probiotics during the study period, we could not further compare the efficacy of different probiotics. We will examine the efficacy of different probiotics in preventing HCC in the future. In addition, the side effects of probiotics for patients were difficult to evaluate in this study. Before probiotic therapy is used to prevent HCC in practice, prospective trials should be conducted to assess its efficacy and safety. Lastly, gut microbiota data were not available in our database; therefore, we did not analyze the association between the microbiota population and HCC risk. We are currently conducting a prospective study using metabonomics, transcriptomics, and proteomic analyses to determine the relationship between the microbiome and carcinogenesis.

## Conclusion

This study demonstrated that probiotics might reduce the risk of HCC occurrence in patients with HBC in a dose-dependent manner. However, further prospective studies are required to confirm the preventive effects of probiotics against HCC.

## Data availability statement

The original contributions presented in the study are included in the article/**Supplementary Material**. Further inquiries can be directed to the corresponding author.

## Ethics statement

The studies involving human participants were reviewed and approved by The Ethical Review Committee of the Beijing Ditan Hospital. Written informed consent for participation was not required for this study in accordance with the national legislation and the institutional requirements.

## Author contributions

XW and KS conceived and designed the project. KS, QZ, YZ, and YB collected the data. KS and QZ analyzed and interpreted the data. KS drafted the manuscript. YZ, YB, and XZ was responsible for manuscript modification. All authors contributed to the article and approved the submitted version.
