# Supplementary material for: Association between probiotic therapy and the risk of hepatocellular carcinoma in patients with hepatitis B-related cirrhosis
Source: Front Cell Infect Microbiol. 2023 Jan 13;12:1104399. doi: 10.3389/fcimb.2022.1104399 (PMC9880196; doi:10.3389/fcimb.2022.1104399)
Supplement: Supplementary file 1 [file Table_1.docx]

**Supplemental Table 1. The detailed information for probiotics in this study.**

| **Probiotics name** | **Pharmacology and toxicology** | **The maintenance dose per day** | **Specifications** | **DDD** |
| --- | --- | --- | --- | --- |
| *Bacillus licheniformis* capsule | This product has antagonistic effect on *Staphylococcus* and *Saccharomycetes*, and promotes the growth of *Bifidobacterium*, *Lactobacillus*, *Bacteroides* and *Peptostreptococcus*. | Two capsules each time, three times a day. | 0.25g (250 million live bacteria). | 1.5 g |
| Live combined *Bacillus subtilis* and *Enterococcus faecium* enteric-coated capsules | This product contains *Enterococcus Faecium* and *Bacillus subtilis*, which can supplement normal physiological live bacteria and inhibit excessive reproduction of harmful bacteria. | Two capsules each time, three times a day. | 0.25g (500 million live bacteria, *Enterococcus Faecium*: 4.5×10^8^, *Bacillus subtilis*:5.0×10^7^). | 1.5g |
| Live combined *Bifidobacterium*, *Lactobacillus*, and *Enterococcus* capsules | This product can directly supplement normal physiological bacteria of human body, regulate the balance of intestinal flora, inhibit pathogenic bacteria in intestinal tract, and reduce the production of enterogenic toxin. | Three capsules each time, two times a day. | 0.21g (The number of viable bacteria is more than 1.0×10^7^ CFU). | 1.26 g |
| Live combined *Bifidobacterium*, *Lactobacillus*, *Enterococcus*, and *Bacillus cereus* tablets | This product contains *Bifidobacterium infantis*, *Lactobacillus acidophilus*, and *Enterococcus faecalis*, which can inhibit pathogenic bacteria in intestinal tract. *Bacillus cere.us* can consume oxygen and create an anaerobic environment for anaerobic bacteria such as *Bifidobacterium*. | Three capsules each time, three times a day. | 0.5g (The number of *Bifidobacterium infantis*, *Lactobacillus acidophilus*, and *Enterococcus faecalis* are more than 0.5×10^6^ CFU; the number of *Bacillus cereus* is more than 0.5×10^5^ CFU). | 4.5 g |

DDD: defined daily dose.
